# Regulation of cell fate determination by single-repeat R3 MYB transcription factors in Arabidopsis

**DOI:** 10.3389/fpls.2014.00133

**Published:** 2014-04-08

**Authors:** Shucai Wang, Jin-Gui Chen

**Affiliations:** ^1^Key Laboratory of Molecular Epigenetics of MOE, Institute of Genetics and Cytology, Northeast Normal UniversityChangchun, China; ^2^Biosciences Division, Oak Ridge National LaboratoryOak Ridge, TN, USA

**Keywords:** CPC, GL1, GL2, GL3, R3 MYB, root hair, trichome, TRY

## Abstract

MYB transcription factors regulate multiple aspects of plant growth and development. Among the large family of MYB transcription factors, single-repeat R3 MYBs are characterized by their short sequence (<120 amino acids) consisting largely of the single MYB DNA-binding repeat. In the model plant Arabidopsis, R3 MYBs mediate lateral inhibition during epidermal patterning and are best characterized for their regulatory roles in trichome and root hair development. R3 MYBs act as negative regulators for trichome formation but as positive regulators for root hair development. In this article, we provide a comprehensive review on the role of R3 MYBs in the regulation of cell type specification in the model plant Arabidopsis.

## Introduction

In plants, MYB transcription factors are encoded by a large family of genes (Stracke et al., 2001; Chen et al., 2006; Dubos et al., 2010; Katiyar et al., 2012). They play important roles in regulating plant growth and development and plant responses to environmental stimuli. There are several sub-families of MYB transcription factors, defined by the number of MYB DNA-binding domain repeats. These include 4R-MYB, 3R-MYB, R2R3-MYB, and 1R-MYB subfamilies that contain four, three, two and one MYB DNA-binding domain repeats, respectively (Dubos et al., 2010).

In the model plant Arabidopsis, there are a total of 64 1R-MYB or MYB-related proteins (Dubos et al., 2010). Among this subfamily, there are a unique set of 1R-MYBs that are characterized by their short sequence (<120 amino acids) consisting largely of the single R3 MYB repeat. These small proteins are referred as single-repeat R3 MYB transcription factors (R3 MYBs) and are subjects of this review article. We provide a comprehensive review about the function and action of R3 MYBs in the model plant Arabidopsis, particularly, in trichome and root hair development.

## R3 MYBs in Arabidopsis

**Figure 1:**
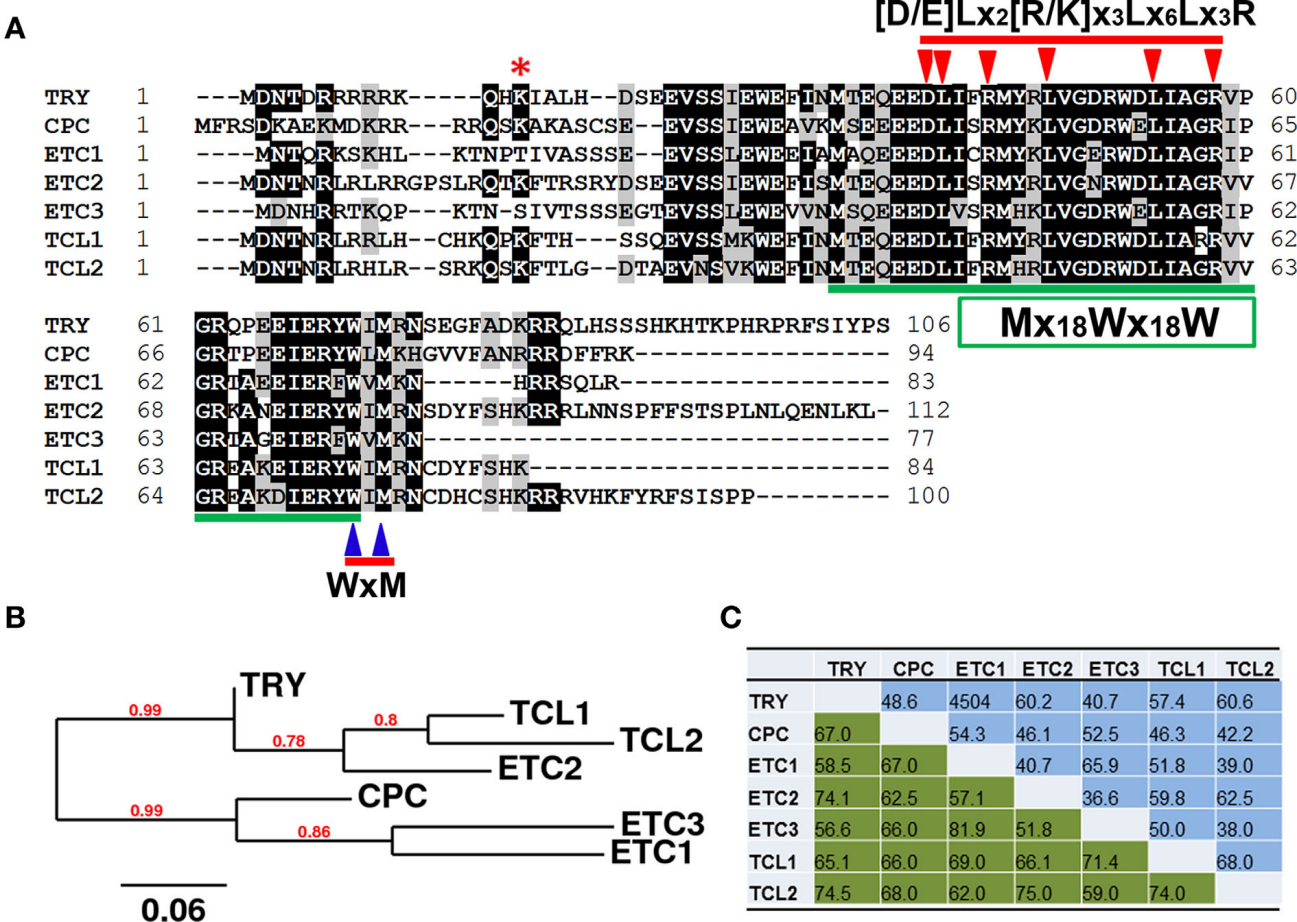
**Single-repeat R3 MYB transcription factors in *Arabidopsis thaliana*. (A)** Amino acid sequence alignment of R3 MYBs. Identical and similar amino acids are shaded in black and gray, respectively. The amino acids of [D/E]Lx_2_[R/K]x_3_Lx_6_Lx_3_R signature that is required for interaction between MYBs and R/B-like bHLH transcription factors are indicated by arrowheads on the top of amino acids. The amino acids of WxM signature that is crucial for cell-to-cell movement are indicated by arrowheads on the bottom of amino acids. The pattern of primary structure of MYB repeat, Mx_18_Wx_18_W, is indicated in a box and amino acids are underlined. The Lysine19 (K19) of ETC2 that was proposed to control its stability is indicated by an asterisk. **(B)** Phylogenetic analysis of R3 MYBs. The full-length amino acid sequences of each R3 MYB were used for the analysis. **(C)** Similarity and identity of amino acid sequences of R3 MYB proteins.

In the completely-sequenced model plant *Arabidopsis thaliana* (hereafter referred as Arabidopsis), a total of seven genes encoding R3 MYBs have been reported so far. These include TRIPTYCHON (TRY)(Schnittger et al., 1999; Schellmann et al., 2002), CAPRICE (CPC) (Wada et al., 1997, 2002), ENHANCER OF TRY AND CPC1 (ETC1) (Esch et al., 2004; Kirik et al., 2004a), ETC2 (Kirik et al., 2004b), ETC3/CAPRICE-LIKE MYB3 (CPL3) (Simon et al., 2007; Tominaga et al., 2008; Wang et al., 2008), TRICHOMELESS1 (TCL1) (Wang et al., 2007) and TCL2/CPL4 (Gan et al., 2011; Tominaga-Wada and Nukumizu, 2012). These seven R3 MYB proteins share approximately 52–82% similarity, and 37–68% identity to each other at the amino acid level (Figure 1). They consist of largely the single MYB DNA-binding repeat, with the total number of amino acid of each protein ranging from 77 to 112.

In order to search for any other potential R3 MYBs encoded by the Arabidopsis genome, the amino acid sequence of TRY was used as a template for searching protein sequence homologs encoded by the fully-sequenced Arabidopsis genome using the “Protein Homologs” tool of Phytozome (www.phytozome.net). In addition to CPC, ETC1, ETC2, ETC3, TCL1 and TCL2, the search identified eight other proteins. These included MYB3, MYB4, MYB5, MYB23, MYB82, GL1, TT2, and WER (Figure S1). However, R3 MYBs were clustered in one distinct branch of the phylogenetic tree (Figure S2). Furthermore, those eight MYBs are mostly R2R3-MYBs, are typically twice the size of R3 MYBs in term of number of amino acids, and share low similarity (17–34%) and identity (10–23%) with R3 MYBs at the amino acid level (Figure S3). Therefore, these MYBs are not considered to be R3 MYB proteins and no additional R3 MYB was identified.

It should be noted that an R3 MYB-related protein, designated as MYBL2, has been shown to be involved in the regulation of flavonoid biosynthesis (Dubos et al., 2008; Matsui et al., 2008). Amino acid alignment and phylogenetic analysis indicated that MYBL2 is more similar to other MYBs than R3 MYBs. For example, the number of amino acid of MYBL2 (195 aa) is almost twice that of R3 MYBs (Figure S1). In addition, the cell-cell movement motif WxM (further discussed in the next section) is not conserved in MYBL2 (Figure S1). Therefore, MYBL2 is not included in our further discussion of R3 MYBs in this review article.

It should also be noted that there are three transcript variants for *ETC3*, designated as *ETC3.1*, *ETC3.2* and *ETC3.3*, according to the current annotation by TAIR (http://www.arabidopsis.org) and Phytozome. Proteins encoded by these three *ETC3* transcript variants are identical except that compared with ETC.1, ETC3.2 has deletion of three amino acids and ETC3.3 has deletion of two amino acids at a site prior to the conserved bHLH binding sequence signature and cell-to-cell movement motif (described in the next section) (Figure S4). For simplicity, only ETC3.1 was included in our analysis.

Finally, several chimeric transcripts can be formed between *ETC2* and *TCL2*/*CPL4*, two tandem repeat genes in Chromosome II (*ETC2*: At2g30420; *TCL2/CPL4*: At2g30424), resulting from alternative splicing (Tominaga-Wada and Nukumizu, 2012).

In summary, the genome of model plant Arabidopsis encodes a total of seven R3 MYBs, and these seven R3 MYBs are the subjects of this review article.

## R3 MYBs structural features

In addition to the small size (77–112 aa) and the constitution of a single MYB repeat, there are a number of structural characteristics of R3 MYBs (Figure 1). Firstly, R3 MYBs contain a sequence signature of [D/E]Lx_2_[R/K]x_3_Lx_6_Lx_3_R that has been shown to be required for the interaction between R3 MYBs and R/B-like bHLH transcription factors (Zimmermann et al., 2004). It should be noted that this bHLH interaction signature is also conserved in many other MYB proteins including those showed sequence homology with R3 MYBs (Figure S1). Secondly, R3 MYBs contain a sequence motif WxM that has been shown to be required for its cell-to-cell movement (Kurata et al., 2005). This cell-to-cell movement motif is not conserved in any other MYBs that showed sequence homology with R3 MYBs (Figure S1). Finally, the primary structure of MYB repeat in R3 MYBs follow a pattern of Mx_18_Wx_18_W, instead of [F/I]x_18_Wx_18_W that is typically found in the R3 MYB repeat of R2R3-MYB proteins and MYBL2 (Figure S1). Therefore, a combination of these three structural features together with the small size and the constitution of a single MYB repeat define a small family of R3 MYB transcription factors.

Due to its constitution of a single MYB repeat, R3 MYBs lack the activation domain that is typically present in most transcription factors. Therefore, from the domain structural perspective, it is believed that R3 MYBs rely on the interaction with other transcription factors to execute their transcriptional activity. As discussed in details below, this indeed represents a major mechanism of action of R3 MYBs.

## Function of R3 MYBs in trichome development

Trichomes are hair cells produced by the outward growth of epidermal cells (Marks, 1997; Hülskamp and Schnittger, 1998). They distribute on the surface of aerial organs including leaves, stems and flower organs of most land plants. Trichomes can act as barriers to protect plants from biotic (e.g., insect herbivores) and abiotic stresses, UV light irradiation and excessive transpiration (Mauricio and Rausher, 1997; Eisner et al., 1998; Werker, 2000; Wagner et al., 2004). Some trichomes or trichome-produced compounds are of great commercial value. For example, single-celled seed trichomes of *Gossypium hirsutum*, cotton fibers, are the most important natural fiber for the textile industry (Kim and Triplett, 2001). Multi-celled glandular trichomes of *Artemisia annua* accumulates artemisinin, a drug that is widely used for the treatment of malaria (Liu et al., 2006; Lommen et al., 2006). Therefore, study of trichome development may lead to the improvement of agronomical and economical traits in plants such as resistance to insect herbivores, improvement of cotton fiber yield and quality, and increase in yield of oil or other plant products contained in glandular trichomes. Furthermore, trichome has become an excellent system to study cell type specification (Schiefelbein, 2003; Pesch and Hülskamp, 2004; Serna, 2005; Schellmann et al., 2007). In this review article, we specifically focus on discussing the role of R3 MYBs in single-celled trichome development in the model plant Arabidopsis.

The genetic control of trichome development has been studied extensively in the last 20 years. It has been generally recognized that trichome initiation is promoted by an activator complex consisting of a WD40-repeat protein, TRANSPARENT TESTA GLABRA1 (TTG1) (Galway et al., 1994; Walker et al., 1999), an R2R3 MYB-type transcription factor, GLABRA1 (GL1) (Oppenheimer et al., 1991), and a bHLH transcription factor, GLABRA3 (GL3) or ENHANCER OF GLABRA3 (EGL3)(Payne et al., 2000; Zhang et al., 2003). Yeast two hybrid experiments showed that both GL1 and TTG1 bind GL3 but at different regions of the GL3 protein and that TTG1 and GL1 do not interact (Payne et al., 2000). This TTG1-GL3/EGL3-GL1 activator complex induces the expression of a homeodomain protein, GLABRA2 (GL2) (Rerie et al., 1994; Masucci et al., 1996), which is required for trichome initiation. The trichome initiation involves feedback loop controls: in addition to inducing *GL2* expression, the TTG1-GL3/EGL3-GL1 activator complex induces the expression of R3 MYB genes. R3 MYBs can move from a trichome precursor cell to its neighboring cell and compete with GL1 for binding GL3 or EGL3 thus disrupting the functionality of the activator complex, resulting in the inhibition of trichome initiation (Hülskamp et al., 1994; Schellmann et al., 2002; Esch et al., 2003; Schiefelbein, 2003; Pesch and Hülskamp, 2004; Ishida et al., 2008; Pesch and Hülskamp, 2009).

According to this model, R3 MYBs act as negative regulators of trichome initiation. Consistent with this view, overexpression of each of seven R3 MYBs resulted in glabrous phenotypes and loss-of-function mutation in R3 MYBs resulted in more trichome formation except *etc1* single mutant (Wada et al., 1997; Schnittger et al., 1999; Schellmann et al., 2002; Wada et al., 2002; Esch et al., 2004; Kirik et al., 2004a,b; Simon et al., 2007; Wang et al., 2007; Tominaga et al., 2008; Wang et al., 2008; Wester et al., 2009; Gan et al., 2011; Tominaga-Wada and Nukumizu, 2012). However, differences in severity and pattern of trichome formation have been observed in the single mutants of R3 MYB genes. The trichome phenotypes of R3 MYB single mutants can be categorized into three groups: (i) increased trichome clustering on leaf (*try*). (ii) increased trichome density on leaf (*cpc*, *etc2*, and *etc3*), and (iii) normal trichome density on leaves but increased trichomes on inflorescence stems and pedicels (*tcl1*, *tcl2*). It should be noted that the phenotype of increased trichome number on leaves of *etc2* and *etc3* single mutants is generally weak, which have resulted in inconsistent results among different studies. For example, the trichome phenotype of *etc2* single mutant was not detected in a few studies (Simon et al., 2007; Tominaga et al., 2008; Wang et al., 2008) but was detected in the study by Kirik et al. (2004b). Analysis of Arabidopsis natural variation population supported that *ETC2* regulates trichome formation on leaves (Hilscher et al., 2009). As mentioned above, among all single mutants of R3 MYB genes, only *etc1* single mutant did not display any detectable trichome phenotypes (Kirik et al., 2004a). Taken together, analysis of single mutants of R3 MYB genes suggested that among seven R3 MYBs, TRY is the predominant member controlling trichome clustering, TCL1 and TCL2 are the predominant members controlling trichome development on inflorescence stem and pedicels, and CPC, ETC2, and ETC3 mainly regulate trichome development on leaves. Table 1 summarizes the trichome and root hair phenotypes of R3 MYB single mutants and overexpression lines.

**Table 1:** **Summary of R3 MYB transcription factors in Arabidopsis**.

	**Full name**	**Locus identifier**	**Phenotype of single mutant**		**Phenotype of overexpressor**		**References**
			**Trichome**	**Root hair**	**Trichome**	**Root hair**	
TRY	TRIPTYCHON	At5g53200	Trichome clusters on leaf	Reduced	Reduced	Increased	Schnittger et al., 1999; Schellmann et al., 2002
CPC	CAPRICE	At2g46410	Increased on leaf	Strongly reduced	Reduced	Increased	Wada et al., 1997, 2002
ETC1	ENHANCER OF TRY AND CPC1	At1g01380	Wild type-like	Wild type-like	Reduced	Increased	Esch et al., 2004; Kirik et al., 2004a
ETC2	ENHANCER OF TRY AND CPC2	At2g30420	Slightly increased on leaf	Wild type-like	Reduced	Increased	Kirik et al., 2004b
ETC3/ CPL3	ENHANCER OF TRY AND CPC3/CAPRICE-LIKE MYB3	At4g01060	Slightly increased on leaf	Slightly reduced	Reduced	Increased	Simon et al., 2007; Tominaga et al., 2008; Wang et al., 2008; Wester et al., 2009
TCL1	TRICHOMELESS1	At2g30432	Increased on upper inflorescence stem and pedicel	Wild type-like	Reduced	Wild type-like	Wang et al., 2007
TCL2/ CPL4	TRICHOMELESS2/CAPRICE-LIKE MYB4	At2g30424	Increased on upper inflorescence stem and pedicel	Wild type-like	Reduced	Wild type-like	Gan et al., 2011; Tominaga-Wada and Nukumizu, 2012

**Figure 2:**
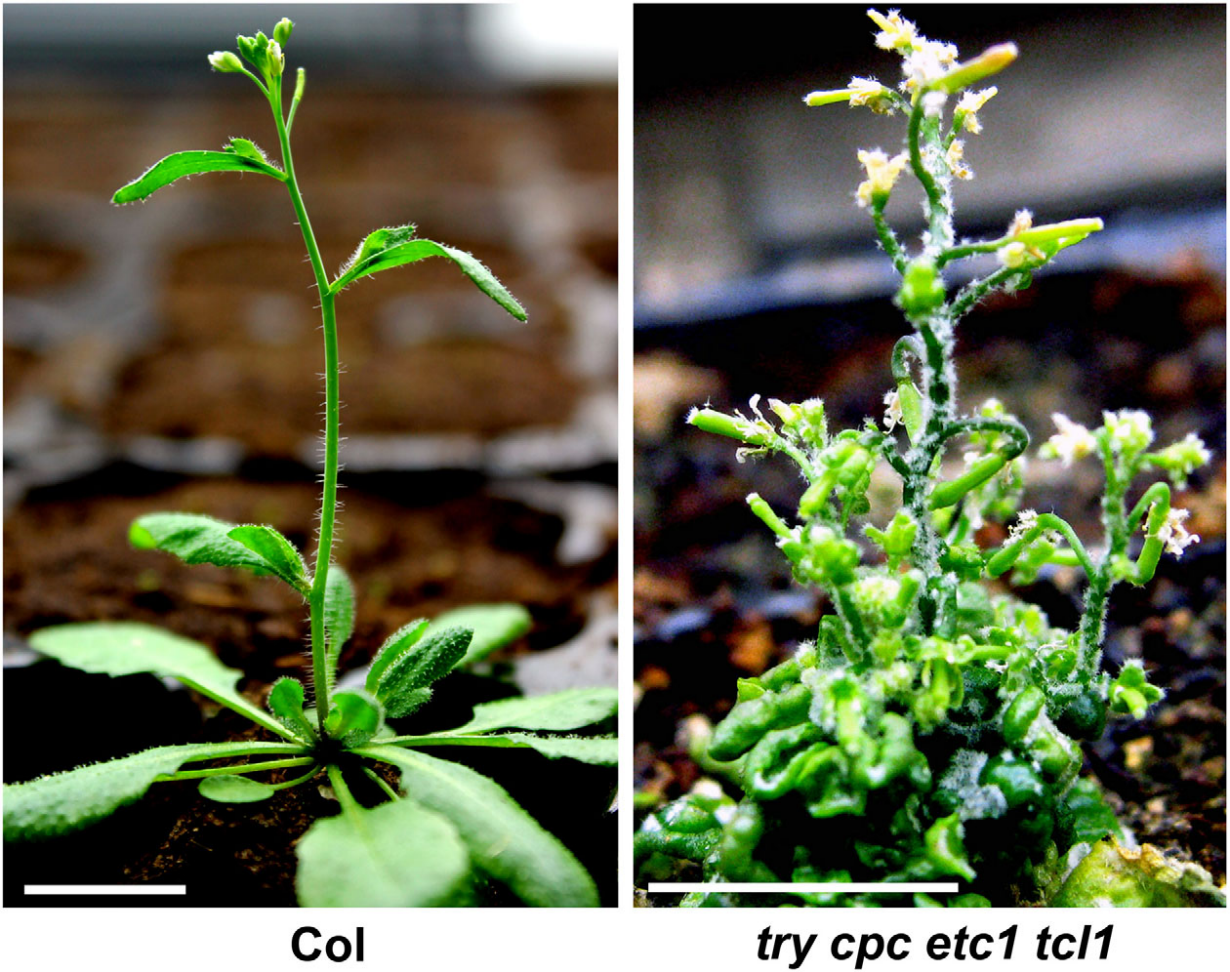
**R3 MYB transcription factors function redundantly to control trichome formation in Arabidopsis**. In Col wild type plant (left), trichomes were observed on leaves, lower part of stems and flower organs. In *try cpc etc1 tcl1* quadruple mutant (right), trichomes were observed in almost all aerial parts of the plants.

Analysis of double, triple, quadruple and higher order mutants revealed redundancy among R3 MYB genes in each of these three categories. For example, *try cpc* double mutants have more trichome clusters than *try* single mutant and have more trichomes on leaves than *cpc* single mutant (Schellmann et al., 2002; Kirik et al., 2004a; Wang et al., 2008). Analysis of *tcl1 cpc etc1 etc3* quadruple mutants revealed that *TCL1* is also involved in the regulation of trichome density on leaves (Wang et al., 2008). Analysis of *tcl1 cpc* double mutants revealed that CPC is also involved in the regulation of trichome formation on upper inflorescence stem and pedicles (Wang et al., 2007). *try cpc etc1 tcl1* quadruple mutants form trichomes in almost all aerial parts of the plant (Wang et al., 2008) (Figure 2). Although *etc1* single mutant did not display any detectable trichome phenotypes, analysis of double, triple and quadruple mutants suggested that *etc1* mutation can enhance trichome phenotypes in each of those three categories (Kirik et al., 2004a; Wang et al., 2008; Wester et al., 2009).

Taken together, all seven R3 MYBs negatively regulate trichome formation in a largely redundant manner. Specificities have also been observed, which will be further discussed in a later section.

## Function of R3 MYBs in root hair development

Root hair is a tubular outgrowth of a trichoblast, a hair-forming cell on the epidermis of a plant root (Gilroy and Jones, 2000). Root hairs are formed in the region of maturation zone of the root. They are lateral extensions of a single cell and rarely branched. Root hairs help plant collect water and mineral nutrients (Gilroy and Jones, 2000). Similar to the trichome system, root hair has become an excellent system to study cell type specification (Ishida et al., 2008; Schiefelbein et al., 2009; Tominaga-Wada et al., 2011; Grebe, 2012; Ryu et al., 2013). Root hair development follows a position-dependent pattern. Epidermal cells in contact with two underlying cortical cells differentiate into hair cells (H cells; trichoblasts) whereas cells that contact only a single cortical cell differentiate into mature hairless cells (N cells; atrichoblasts) (Schiefelbein, 2000; Dolan and Costa, 2001).

The genetic control of root hair formation is remarkably similar to that of trichome initiation with all same components in the activator complex except that GL1 is replaced by another R2R3-MYB transcription factor, WEREWOLF (WER) (Lee and Schiefelbein, 1999). Recently, it was found that WER positively regulates the expression of *MYB23*, an R2R3 MYB, during root epidermis development (Kang et al., 2009). Similar to WER, MYB23 specifies non-hair cell type and can substitute for the function of WER. Unlike WER, MYB23 regulates its own expression, providing a positive feedback loop to reinforce cell fate decisions and root epidermal patterning (Kang et al., 2009). The TTG1-GL3/EGL3-WER activator complex induces the expression of *GL2* and *R3 MYBs*. R3 MYBs can move from an N cell to a neighboring H cell to compete with WER for binding GL3 or EGL3, thus limiting the activity of the TTG1-GL3/EGL3-WER activator complex (Ishida et al., 2008; Schiefelbein et al., 2009; Tominaga-Wada et al., 2011; Grebe, 2012; Ryu et al., 2013). Opposing to that in the trichome initiation system, positive regulators of trichome initiation function as negative regulators of root hair formation and vice versa. Therefore, R3 MYBs are positive regulators of root hair formation.

According to this model, overexpression of R3 MYBs is expected to result in increased root hair formation whereas loss-of-function mutation results in decreased root hair formation. This is indeed the general trend. However, differences have been observed. TCL1 and TCL2 appeared to have diverged the most among R3 MYB subfamily because overexpression of TRY, CPC, ETC1, ETC2, and ETC3 rendered more root hair formation (Tominaga et al., 2008) whereas overexpression of TCL1 and TCL2 had no effect on root hair formation (Wang et al., 2007; Gan et al., 2011). Among all single mutants of R3 MYBs, only *try* and *cpc* single mutants have been consistently shown to have decreased root hair formation and *cpc* single mutant had stronger root hair phenotype than *try* mutant (Wada et al., 1997, 2002; Schellmann et al., 2002). Although *etc3* single mutant was shown to display wild-type root hair phenotypes in two studies (Simon et al., 2007; Wang et al., 2008), it was shown to produce less root hairs in two other studies (Tominaga et al., 2008; Wester et al., 2009), indicating that the root hair phenotype of *etc3* mutant is generally weak. Other single mutants including *etc1*, *etc2*, *tcl1*, and *tcl2* displayed little or no root hair phenotypes. These results suggested that CPC is the predominant member of R3 MYB family controlling root hair formation.

Analysis of double, triple and quadruple mutants revealed that other R3 MYB genes may function redundantly with CPC to positively regulate root hair formation. Analysis of *etc1 cpc* double mutant and *etc1 try cpc* triple mutant revealed that *ETC1* functioned redundantly with *TRY* and *CPC* to control root hair formation (Kirik et al., 2004a). Analysis of *cpc etc1 etc3 tcl1* quadruple mutants indicated that *TCL1* positively regulate root hair formation (Wang et al., 2008). Analysis of *etc3 cpc* double mutant revealed that *ETC3* functioned redundantly with *CPC* to control root hair formation (Tominaga et al., 2008). Taken together, five of the seven R3 MYB genes have been shown to be involved in the regulation of root hair formation. So far, a role of ETC2 and TCL2 in root hair formation has not been reported.

## Other functions of R3 MYBs

In addition to regulating trichome and root hair development, R3 MYBs have been shown to be involved in the regulation of other processes including flowering, anthocyanin accumulation and stomatal formation. This is consistent with the observation that the expression of R3 MYB genes is not restricted to trichomes and root hairs. For example, *TRY* is also expressed in inflorescence and siliques (Schellmann et al., 2002).

*etc3/cpl3* mutant was shown to flower earlier with fewer leaves than the wild-type (Tominaga et al., 2008; Tominaga-Wada et al., 2013b) although the early flowering phenotype was not reproduced by another study (Wester et al., 2009). Subsequently, it was shown that mutations in *TRY* or *CPC* delayed flowering of *etc3*/*cpl3* mutant and a mutation in *ETC1* did not further delay flowering but reduced plant size (Tominaga-Wada et al., 2013b). Mutations in ETC3/CPL3 were also shown to affect endoreduplication (Tominaga et al., 2008).

CPC was found to be a positive regulator of stomatal formation in the hypocotyl (Serna, 2008). CPC was localized in the nucleus and peripheral cytoplasm of fully differentiated epidermal cells in hypocotyl. *CPC* expression in differentiating stomaless-forming cells was shown to be positively regulated by TOO MANY MOUTHS (TMM), a leucine-rich repeat-containing receptor-like protein expressed in proliferative postprotodermal cells (Nadeau and Sack, 2002). Furthermore, *CPC* acts redundantly with *TRY* to promote stomata formation.

Because GL3, EGL3, and TTG1 of the activator complex are also involved in the regulation of seed coat mucilage and anthocyanin production, in addition to the regulation of trichome and root hair formation (Zhang et al., 2003), and that TTG1-GL3/EGL3-GLl and TTG1-GL3/EGL3-WER complexes activate the expression of R3 MYB genes, it raises a question of whether R3 MYBs also regulate anthocyanin and seed coat mucilage production. Molecular and genetic studies suggested that R3 MYBs regulate anthocyanin biosynthesis but not seed coat mucilage production.

CPC was shown to be a negative regulator of anthocyanin biosynthesis (Zhu et al., 2009). Overexpression of *CPC* repressed a total of 85 genes at the whole genome level. Of these 85 genes, seven are later anthocyanin biosynthesis genes. As discussed above, the action of R3 MYBs in the regulation of trichome and root hair development involves the TTG1-GL3/EGL3-GL1 and TTG1-GL3/EGL3-WER activator complex, respectively. The regulation of anthocyanin biosynthesis involves a similar TTG1-bHLH-MYB activator complex (Walker et al., 1999; Zhang et al., 2003; Zimmermann et al., 2004; Gonzalez et al., 2008). Unlike the trichome and root hair activator complexes, the MYB function is executed by PAP1 and PAP2. GL3/EGL3 interacts with both TTG1 and PAP1/PAP2, acting as positive regulators of anthocyanin biosynthesis (Zhang et al., 2003; Zimmermann et al., 2004). Transient expression analysis indicated that CPC competes with PAP1/PAP2 for binding with GL3/EGL3. Similarly, a tomato ortholog of TRY was also shown to negatively regulate anthocyanin accumulation (Nukumizu et al., 2013). Among all R3 MYBs, only CPC appears to act in the negative feedback on anthocyanin accumulation in response to nitrogen starvation conditions (Nemie-Feyissa et al., 2014).

For seed coat mucilage, in addition to TTG1, it requires bHLH transcription factor EGL3 and TT8, and MYB transcription factor MYB61 (Zhang et al., 2003). Analysis of single, double and higher order R3 MYB mutants did not reveal defects in seed mucilage production (Zhu et al., 2009; Wang et al., 2010), suggesting that R3 MYBs do not regulate seed mucilage biosynthesis.

## Redundancy and specificity of R3 MYBs

As discussed above, R3 MYBs can function redundantly to regulate trichome and root hair development. However, although R3 MYBs are small in size (77–112 aa) and are similar (52–82%) to each other at the amino acid level, some members exhibit distinct function than others in trichome and root hair development. For example, among all R3 MYBs, TRY is characteristic of its regulatory role in trichome clustering, CPC is characteristic of its role in root hair development, and TCL1 and TCL2 are characteristic of their roles in trichome development on the inflorescence stem and pedicels. What determines the functional specificity of each R3 MYB? In general, there are a number of attributes of functional specificity of any given genes including the expression level, spatiotemporal expression of the gene and the biochemical property of the protein (e.g., transcriptional activity, protein stability and protein subcellular localization). The expression level and pattern of a gene are largely determined by the promoter activity whereas the biochemical properties of the protein are determined by the amino acid constitution of the protein. R3 MYBs display differences in both categories.

### Promoter activity

At the transcript level, differences in tissue/organ expression of R3 MYB genes have been observed. Among all seven R3 MYB genes, *ETC2* and *TCL1* were not expressed in the root (Kirik et al., 2004b; Wang et al., 2007). The transcript of *TCL2/CPL4* was also not detected or at very low level in the root as determined by RT-PCR (Gan et al., 2011; Tominaga-Wada and Nukumizu, 2012). These results implied that these three R3 MYB genes do not play a major role in regulating root hair formation. This view is consistent with the observation that none of *etc2*, *tcl1*, and *tcl2* single mutants displayed root hair phenotypes. Therefore, the differences in tissue/organ expression patterns of R3 MYBs determine the first level of specificities. Nonetheless, all of these three genes tested were able to partially rescue the root hair phenotype of *cpc* mutant when their expression was driven by *CPC* promoter (Simon et al., 2007). These results reinforce the concept that the normal functions of these genes are restricted by their promoter activities.

Results from promoter-swap experiments supported that transcriptional regulation is important for the functional diversity of R3 MYB genes (Wester et al., 2009). When *ETC3* was expressed under the promoter of *ETC3*, *TRY* or *CPC*, it could equally rescue the trichome phenotype of *etc3* mutant (Wester et al., 2009), suggesting that the promoters of *TRY*, *CPC*, and *ETC3* are interchangeable with respect to *etc3* single mutant rescue. However, when these constructs (*pETC3:ETC3*, *pTRY:ETC3*, and *pCPC:ETC3*) were used to complement triple mutants containing *etc3*, qualitative differences were observed. *pETC3:ETC3* construct rescued the *cpc try etc3* mutant to the same extent as the *try cpc* mutant whereas *pTRY:ETC3* and *pCPC:ETC3* displayed an over-rescued phenotype resembling that of *try*. Differences were also observed between *pTRY:ETC3* and *pCPC:ETC3* in terms of trichome cluster phenotypes. These results suggest that the regulation of expression of R3 MYB genes is important for their functioning.

### Protein properties

*TRY* and *CPC* displayed indistinguishable expression patterns in leaves (Schellmann et al., 2002), yet the trichome phenotypes of *try* and *cpc* single mutants are very different. These suggested that the amino acid sequences of R3 MYBs play important roles in determining its functionality. The importance of protein properties in determining its functionality has been supported by genetic complementation studies, in particularly promoter swapping assays. For example, when *TCL1* was expressed under the control of *TRY* or *CPC* promoter in *try* and *cpc* mutant background, respectively, *TCL1* was only able to partially rescue the trichome and root hair phenotypes of *try* and *cpc* mutants (Wang et al., 2007), indicating that TCL1 does not function equivalently with TRY or CPC. On the other hand, the expression of *TRY* under the control of *TRY* or *CPC* promoter could completely rescue the trichome clustering phenotype of *try* mutant whereas the expression of *CPC* under *TRY* promoter could not (Pesch and Hülskamp, 2011), suggesting that the specific role of *TRY* in regulating trichome cluster formation is not based on its transcriptional regulation but on specific protein properties. Furthermore, it has been suggested that TRY protein has specific properties relevant in the context of both cluster formation and trichome density (Pesch and Hülskamp, 2011).

Differences in R3 MYB protein properties have also been observed in other studies. For example, by using promoter-swap experiments, Simon et al. (2007) compared the ability of several R3 MYBs to rescue the root hair phenotype of *cpc* mutant under *CPC* promoter. It was found that ETC1 possessed the best ability, followed by ETC3, TRY, and ETC2, revealing differences in protein properties.

Taken together, the functional specificity of each R3 MYB was determined both by the promoter activity of each gene and the biochemical property of each protein. Which aspect of protein properties do R3 MYBs differ? The precise answer for this question is unclear. Recent studies, however, have revealed some possibilities.

#### Binding strength to GL3/EGL3

During trichome patterning, two important actions of R3 MYBs at the protein level are the competition with GL1 for bindings GL3 and the movement from trichome cell to its neighboring cells. The transcriptional activity and their binding strength with bHLH transcription factors (GL3/EGL3) of R3 MYBs have been studied in the yeast system and the bi-molecular fluorescence complementation (BiFC) system (Wester et al., 2009), and in the Arabidopsis mesophyll protoplast transient expression system (Wang et al., 2008). Although these assays could not distinguish differences in protein stability, these studies indicate that differences in biochemical property do exist among members of R3 MYBs. It was found that R3 MYBs including TRY, CPC, ETC1, ETC2, and ETC3 differ in their binding strength to GL3 (Wester et al., 2009). Furthermore, by using the yeast three-hybrid system, it was found that R3 MYBs' capacity to compete with GL1 for binding to GL3 also differs with CPC being the most potent inhibitor followed by ETC1, TRY, ETC3, and ETC2 (Wester et al., 2009). Such differences in competing GL1 for binding GL3 may contribute to the functional specificities of R3 MYBs.

#### Cell-to-cell movement

The cell-to-cell movement motif is conserved in all R3 MYB proteins, and the cell-to-cell movement has been experimentally demonstrated for CPC and ETC3 (Kurata et al., 2005; Wester et al., 2009). It has been shown that CPC is able to move readily within the root epidermis when its expression level is high (Kang et al., 2013). CPC is capable of moving from the stele tissue in the center of the root to the outermost epidermal layer, where it can induce the hair cell fate (Kang et al., 2013). The accumulation and localization of CPC in the nuclei of H-position cells require EGL3. During trichome patterning, it was found that ETC3 protein was localized in the nucleus as well as in the cytoplasm in trichome initials whereas ETC3 protein was restricted to the nucleus in surrounding cells (Wester et al., 2009), indicating that ETC3 protein likely moves from the trichome initial into the neighboring epidermal cells. The cell-cell movement of ETC3 was further confirmed using the particle bombardment in single leaf epidermal cells (Wester et al., 2009). Mathematical analysis suggests that the mobility of the inhibitors depends on their affinity for GL3 and predicted that ETC3 moves faster than CPC (Wester et al., 2009). This prediction was experimentally validated in particle bombardment experiments (Wester et al., 2009). Therefore, the difference in cell-to-cell movement ability may also contribute to the function specificities of R3 MYB proteins.

#### Protein subcellular localization

At the protein level, all R3 MYBs are localized in the nucleus as expected for transcription factors but several R3MYB proteins such as TCL1 (Wang et al., 2007), CPC (Serna, 2008) and ETC3 (Wester et al., 2009) are also found to be localized to a site around plasma membrane. Recently, it has been found that subcellular localization of R3 MYBs and GL1 affects their functionality (Pesch et al., 2013). AtMYC1, a homolog of GL3 and EGL3, can regulate the intracellular localization of GL1, TRY and CPC. AtMYC1 can relocate GL1 from the nucleus into the cytoplasm and can be recruited into the nucleus by TRY and CPC. It was suggested that AtMYC1 represses the activity of TRY and CPC (Pesch et al., 2013). It remains unclear to what extent, differences in protein subcellular location of R3 MYBs may affect their functionalities.

#### Protein stability

It is known that the degradation of GL3 and EGL3 proteins is ubiquitin/26S proteasome-dependent (Patra et al., 2013). However, no study has been conducted to directly compare the protein stability of R3 MYBs though it was suggested that a mutation at Lysine19 (K19) of ETC2 may affect its protein stability (Hilscher et al., 2009). This amino acid is also conserved in TRY, CPC, TCL1, and TCL2, but not in ETC1 and ETC3 (Figure 1). It is unclear whether such a difference may contribute to the function specificities of R3 MYB proteins

## Regulation of expression of R3 MYB genes

As discussed above, the trichome and root hair initiation involves feedback loop controls. In addition to inducing *GL2* expression, the TTG1-GL3/EGL3-GL1 (in trichome initiation) and TTG1-GL3/EGLs-WER (in root hair formation) activator complexes also induce the expression of R3 MYB genes. Consistent with these modes, *CPC* has been identified as a direct target gene for WER (Koshino-Kimura et al., 2005; Ryu et al., 2005). WER protein binds three sites in the *CPC* promoter region, designated *WBSI*, *WBSII/CPCMBSI* and *CPCMBSII*, and regulates its transcription. Furthermore, both GL1 and GL3 have been shown to be recruited to the promoter region of *CPC* and *ETC1* (Morohashi et al., 2007; Zhao et al., 2008).

Recent studies suggested the expression of R3 MYB gene may also be controlled by other mechanisms. By using an Arabidopsis protoplast transient expression system, it was found that co-transfection of GL1 or WER, with GL3 or EGL3, is required and sufficient to activate *TRY*, *CPC*, *ETC1*, and *ETC3*, but not *TCL1*, *TCL2* or *ETC2* (Wang et al., 2008; Gan et al., 2011). Furthermore, *MIR156*-regulated SPLs (SQUAMOSA PROMOTER BINDING PROTEIN-LIKE), which are known to play important roles in regulating phase transition and flowering, have been shown to be able to directly activate the expression of *TCL1* and *TRY* through binding to their promoters and that this activation is independent of GL1 (Yu et al., 2010). Reduced expression of *TCL2* in *35S:MIR156* transgenic plants suggested that *MIR156*-targeted SPLs may also regulate the expression of *TCL2* (Gan et al., 2011).

Finally, the proposed regulatory feedback loop between the activators and the inhibitors can also lead to an auto-repression of the inhibitors. Specifically, binding of R3 MYB to GL3 can displace GL1 thereby inactivating the activator complex by forming a different protein complex through the replacement of GL1 with R3 MYBs. Consequently, this creates a shortcut of the regulatory feedback and results in the repression of R3 MYB own expression.

## Modes of action of R3 MYBs

**Figure 3:**
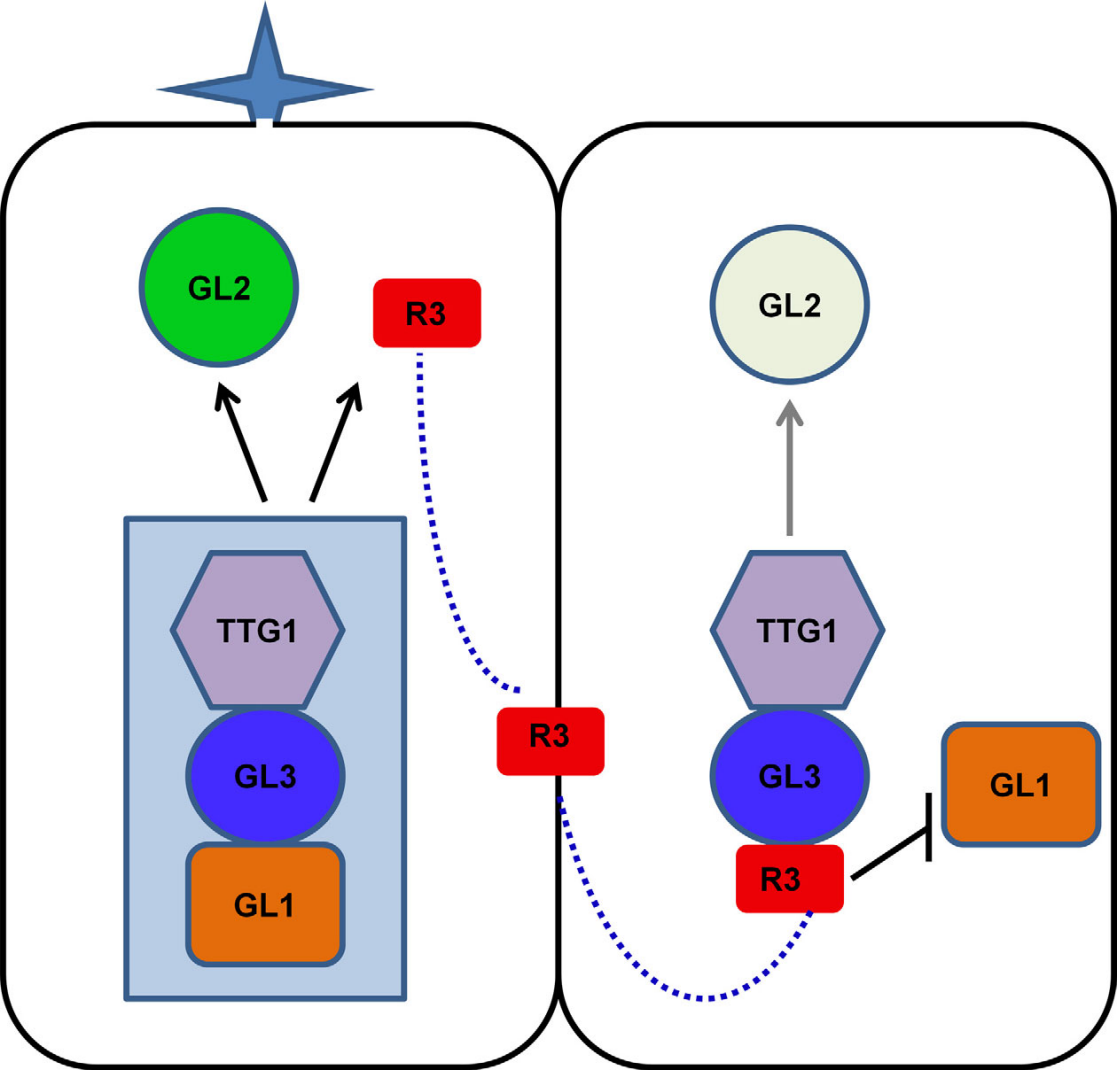
**Model of action of R3 MYB transcription factors in controlling trichome formation**. TTG1, GL3/EGL3, and GL1 form an activator complex to regulate the transcription of *GL2* and R3 MYB genes. R3 MYBs can move from a trichome precursor cell (left) to its neighboring cell (right) to compete with GL1 for binding GL3 or EGL3, thus limiting the activity of the TTG1-GL3/EGL3-GL1 activator complex and resulting in decreased expression of *GL2*. In addition, some R3 MYBs, such as TCL1 and TCL2, can suppress the transcription of *GL1*. These events create differences between cells and ultimately result in a pattern of trichome and non-trichome cells. For simplicity, EGL3 is not shown in the model. R3, R3 MYBs.

As discussed above, the mode of action of R3 MYBs in trichome and root hair development is very similar. The action of R3 MYBs in root hair patterning has been discussed in several recent review articles (Ishida et al., 2008; Schiefelbein et al., 2009; Tominaga-Wada et al., 2011; Grebe, 2012; Ryu et al., 2013; Schiefelbein et al., 2014). To avoid redundancy, here we only focus on describing the action of R3 MYBs in trichome development. Genetic and molecular studies suggested that R3 MYBs move from a trichome precursor cell to its neighboring cell to compete with GL1 for binding GL3 or EGL3, thus limiting the activity of the TTG1-GL3/EGL3-GL1 activator complex and resulting in decreased expression of *GL2*; this results in the inhibition of trichome formation (Figure 3). During trichome patterning, regulatory feedback loops include several important events: (1) the activation of the inhibitors by the activators, (2) the movement of inhibitors between cells, (3) the repression of the activators by the inhibitors, and (4) the auto-repression of the inhibitors. These events created differences between cells and ultimately result in a pattern of trichome and non-trichome cells. This model of action of R3 MYBs is supported both by the binding between R3 MYBs and GL3 and the cell-to-cell movement of MYBs. For example, all seven R3 MYBs have been shown to interact with GL3 in Arabidopsis protoplasts (Wang et al., 2008; Gan et al., 2011). CPC and ETC3 have been shown to be able to move from cell to cell (Kurata et al., 2005; Wester et al., 2009). Although the cell-to-cell movement of other R3 MYBs has not been experimentally demonstrated, the cell-to-cell movement motif is conserved in all seven R3 MYBs (Figure 1).

Recent studies revealed that R3 MYBs also use other distinctive mechanisms to regulate trichome development. Both TCL1 and TCL2 can directly suppress the transcription of *GL1* (Wang et al., 2007; Gan et al., 2011), a member of the TTG1-GL3/EGL3-GL1 activator complex. Chromatin immunoprecipitation assay indicated that TCL1 or its protein complex can directly bind the cis-acting elements that are required for the proper expression of *GL1* (Wang et al., 2007). Overexpression of TCL1 resulted in suppression of *GL1* and loss-of-function of TCL1 resulted in elevated expression of *GL1* (Wang et al., 2007). Thus, some R3 MYBs, such as TCL1 and TCL2, have dual roles in disrupting the functionality of the activator complex: competing with GL1 for binding GL3 and suppressing the expression of *GL1*. The suppression of *GL1* expression by R3 MYBs provides an additional regulation loop to control the activity of activator complex. However, it is unknown if this represent a general mechanism for all members of R3 MYBs. It has been shown that overexpression of *CPC* could also drastically suppress the expression of *GL1* (Wang et al., 2007), suggesting other members of R3 MYB family could also function in a similar manner as TCL1 to directly suppress the transcription of *GL1*. However, it remains elusive whether such a suppression mechanism may also operate similarly during root hair patterning because overexpression of *CPC* was not able to change either the expression pattern or expression level of *WER* in roots (Song et al., 2011).

Cell fate in both trichome and root hair development is determined by competition between positive regulators and negative regulators. For both systems, models explaining pattern formation are similar. These models are largely based on a feedback loop in which the positive regulators activate the negative regulators and the negative regulators inhibit the activators, with the negative regulators being able to move between cells. Although the same machinery is central to the spatial regulation of root hair and trichome patterning, the context is different. Trichome formation can occur without a recognizable reference to other structures except for other trichomes whereas root hair patterning is position-dependent with root hairs normally form only in epidermal cells overlying a cleft between two underlying cortex cells. Such a position-dependent pattern is dependent on a leucine-rich repeat receptor-like kinase SCRAMBLED (SCM) which inhibits *WER* expression in the H cell position (Kwak et al., 2005; Kwak and Schiefelbein, 2007).

Since both *GL2* and R3 MYB genes are activated by the same TTG1-GL3/EGL3-GL1 activator complex but having opposite roles in trichome formation, one might wonder the relationship between GL2 and R3 MYBs in regulating trichome formation. One genetic approach answering this question is to analyze double mutants and higher order mutants between *gl2* and R3 MYB mutants. By doing that, it was found that R3 MYBs could still negatively regulate trichome formation in a redundant manner in *gl2* mutant background (Wang et al., 2010). These studies suggested that R3 MYBs could control trichome formation in a GL2-independent manner. The implication of these studies is that trichome formation may require the expression of other genes in addition to *GL2*. These studies called for additional models for the regulation of trichome development.

## Concluding remarks and future directions

R3 MYBs are best characterized for their regulatory roles in trichome and root hair development. In the last two decades, great progress has been made to elucidate the molecular mechanism of action of R3 MYBs in cell type specification. However, several questions remained to be answered. Firstly, the regulation of expression of R3 MYB genes needs further exploration. Not all members of R3 MYB genes are regulated by the activator complex. Only the expression of *TRY*, *CPC*, *ETC1*, and *ETC3* were found to be regulated by the TTG1-GL3/EGL3-GL3 activator complex. The expression of *TCL1*, *TCL2*, and *TRY* can be regulated by *MIR156*-mediated SPLs. Little is known about how the expression of *ETC2* is regulated. When tested in protoplasts, none of the SPLs tested could activate *Gal4:GUS* reporter gene when recruited to the *Gal4* promoter by a Gal4 DNA binding domain, suggesting that SPLs may need co-activator(s) to activate the transcription of R3 MYB genes (Gan et al., 2011). Secondly, the molecular mechanism of action of R3 MYBs in regulating trichome formation needs to be investigated further. It has been previously thought that R3 MYBs inhibit trichome formation via their competition with GL1 for binding GL3/EGL3, thus limiting the activity of the TTG1-GL3/EGL3-GL1 activator complex and resulting in the down regulation of *GL2*. Now it is known some R3 MYBs, such as TCL1 and TCL2, also directly suppress the expression of *GL1*, which also results in the down regulation of *GL2* that is required for trichome formation. Adding another layer of complexity, recent results suggested R3 MYBs may regulate trichome formation in a GL2-independent manner (Wang et al., 2010). Therefore, the relationship between GL2 and R3 MYB genes in the regulation of trichome formation deserves further investigation. A comprehensive systems approach integrating genetic, genomic and computational analyses would be essential for dissecting complex regulatory networks (Bruex et al., 2012). Thirdly, the movement of R3 MYBs and components of activator complex deserves further investigation. Mathematical model suggested that the movement of both R3 MYBs and GL3 are important for root epidermal patterning (Savage et al., 2008). However, discrepancies in the movement of components of activator complex have been observed. In roots, it was reported that GL3 protein moves from the hair cells to the non-hair cells (Bernhardt et al., 2005). However, in a particle bombardment experiment using GFP fusion proteins, it was found that TRY and CPC, but not GL1 or GL3, can move between cells (Digiuni et al., 2008). In a similar particle bombardment experiment, it was shown that none of TTG1, GL3, GL1 or GL2 moves between adjacent epidermal cells whereas CPC moves to neighboring cells (Zhao et al., 2008). Furthermore, it is unclear whether the cell-to-cell movement of R3 MYB proteins is directional. If so, what controls the directional movement of R3 MYB proteins? A recent study has shed lights on the mechanism of possible directional cell-to-cell movement of R3 MYB proteins (Kang et al., 2013). Specifically, CPC is preferentially expressed in non-hair cells but acts in the root-hair cells. This directional cell-to-cell movement of CPC involves EGL3 which traps CPC protein in the root hair cells (Kang et al., 2013). Fourthly, trichome and root hair patterning involve both negative and positive feedback within and between cells. Much is known about the negative feedback mechanism in which R3 MYBs disrupt the functionality of TTG1-bHLH-MYB activator complex but less is known about the positive feedback. In root hair patterning, MYB23 functions in the positive feedback loop which can substitute the function of WER and bind to its own promoter (Kang et al., 2009). Such a positive feedback can reinforce cell fate decision and ensure robust establishment of the cell type pattern. Finally, R3 MYBs are wildly distributed in plant kingdom. It would be of great interest to examine if they regulate trichome formation in other plant species in a similar manner as that in Arabidopsis. Emerging evidence supports that this is likely the case (e.g., Tominaga-Wada et al., 2013a). Trichomes in many plant species (e.g., cotton fiber) are of great economic values. Manipulation of R3 MYBs expression can potentially contribute to the increase of productivity and quality in economic crops.

### Conflict of interest statement

The authors declare that the research was conducted in the absence of any commercial or financial relationships that could be construed as a potential conflict of interest.

## Supplementary material

Figure S1 **Amino acid sequence alignment of R3 MYBs with other MYBs**. Identical and similar amino acids are shaded in black and gray, respectively. The amino acids of [D/E]Lx_2_[R/K]x_3_Lx_6_Lx_3_R signature that is required for interaction between MYBs and R/B-like bHLH transcription factors are indicated by red arrowheads on the top of amino acids. The amino acids of WxM motif that is crucial for cell-to-cell movement are indicated by blue arrowhead on the top of amino acids. The pattern of primary structure of MYB repeat, Mx_18_Wx_18_W, is indicated by arrows on the bottom of amino acids.

Figure S2 **Phylogenetic analysis of R3 MYBs with other MYBs**. The entire amino acid sequences of single repeat R3 MYB transcription factors were used for phylogenetic analysis using tool on www.phylogeny.fr with default settings.

Figure S3 **Amino acid similarity and identity of R3 MYBs and other MYBs**.

Figure S4 **Amino acid sequence alignment of ETC3 variants**. Identical and similar amino acids are shaded in black and gray, respectively. The amino acids of [D/E]Lx_2_[R/K]x_3_Lx_6_Lx_3_R signature that is required for interaction between MYBs and R/B-like bHLH transcription factors are indicated by arrowheads on the top of amino acids. The amino acids of WxM motif that is crucial for cell-to-cell movement are indicated by arrowheads on the bottom of amino acids.

## Abbreviations

CPC: CAPRICE
CPL3: CAPRICE-LIKE MYB3
CPL4: CAPRICE-LIKE MYB4
EGL3: ENHANCER OF GLABRA3
ETC1: ENHANCER OF TRY AND CPC1
ETC2: ENHANCER OF TRY AND CPC2
ETC3: ENHANCER OF TRY AND CPC3
GL1: GLABRA1
GL2: GLABRA2
GL3: GLABRA3
TCL1: TRICHOMELESS1
TCL2: TRICHOMELESS2
TRY: TRIPTYCHON
TTG1: TRANSPARENT TESTA GLABRA1.

## References

[B1] Bernhardt C. Zhao M. Gonzalez A. Lloyd A. Schiefelbein J. (2005). The bHLH genes GL3 and EGL3 participate in an intercellular regulatory circuit that controls cell patterning in the Arabidopsis root epidermis. Development 132, 291–298 10.1242/dev.01565 15590742

[B2] Bruex A. Kainkaryam R. M. Wieckowski Y. Kang Y. H. Bernhardt C. Xia Y. (2012). A gene regulatory network for root epidermis cell differentiation in Arabidopsis. PLoS Genet. 8:e1002446 10.1371/journal.pgen.1002446 22253603 PMC3257299

[B3] Chen Y. Yang X. He K. Liu M. Li J. Gao Z. (2006). The MYB transcription factor superfamily of Arabidopsis: expression analysis and phylogenetic comparison with the rice MYB family. Plant Mol. Biol. 60, 107–124 10.1007/s11103-005-2910-y 16463103

[B4] Digiuni S. Schellmann S. Geier F. Greese B. Pesch M. Wester K. (2008). A competitive complex formation mechanism underlies trichome patterning on Arabidopsis leaves. Mol. Syst. Biol. 4, 217 10.1038/msb.2008.54 18766177 PMC2564731

[B5] Dolan L. Costa S. (2001). Evolution and genetics of root hair stripes in the root epidermis. J. Exp. Bot. 52, 413–417 10.1093/jexbot/52.suppl_1.413 11326047

[B6] Dubos C. Le Gourrierec J. Baudry A. Huep G. Lanet E. Debeaujon I. (2008). MYBL2 is a new regulator of flavonoid biosynthesis in *Arabidopsis thaliana*. Plant J. 55, 940–953 10.1111/j.1365-313X.2008.03564.x 18532978

[B7] Dubos C. Stracke R. Grotewold E. Weisshaar B. Martin C. Lepiniec L. (2010). MYB transcription factors in Arabidopsis. Trends Plant Sci. 15, 573–581 10.1016/j.tplants.2010.06.005 20674465

[B8] Eisner T. Eisner M. Hoebeke E. R. (1998). When defence backfires: detrimental effect of a plant's protective trichomes on an insect beneficial to the plant. Proc. Natl. Acad. Sci. U.S.A. 95, 4410–4414 10.1073/pnas.95.8.4410 9539750 PMC22502

[B9] Esch J. J. Chen M. A. Hillestad M. Marks M. D. (2004). Comparison of *TRY* and the closely related *At1g01380* gene in controlling Arabidopsis trichome patterning. Plant J. 40, 860–869 10.1111/j.1365-313X.2004.02259.x 15584952

[B10] Esch J. J. Chen M. Sanders M. Hillestad M. Ndkium S. Idelkope B. (2003). A contradictory GLABRA3 allele helps define gene interactions controlling trichome development in Arabidopsis. Development 130, 5885–5894 10.1242/dev.00812 14561633

[B11] Galway M. E. Masucci J. D. Lloyd A. M. Walbot V. Davis R. W. Schiefelbein J. W. (1994). The *TTG* gene is required to specify epidermal cell fate and cell patterning in the Arabidopsis root. Dev. Biol. 166, 740–754 10.1006/dbio.1994.1352 7813791

[B12] Gan L. Xia K. Chen J. G. Wang S. (2011). Functional characterization of TRICHOMELESS2, a new single repeat R3 MYB transcription factor in the regulation of trichome patterning in Arabidopsis. BMC Plant Biol. 11:176 10.1186/1471-2229-11-176 22168948 PMC3264604

[B13] Gonzalez A. Zhao M. Leavitt J. M. Lloyd A. M. (2008). Regulation of the anthocyanin biosynthetic pathway by the TTG1/bHLH/Myb transcriptional complex in Arabidopsis seedlings. Plant J. 53, 814–827 10.1111/j.1365-313X.2007.03373.x 18036197

[B14] Grebe M. (2012). The patterning of epidermal hairs in Arabidopsis–updated. Curr. Opin. Plant Biol. 15, 31–37 10.1016/j.pbi.2011.10.010 22079786

[B15] Hilscher J. Schlötterer C. Hauser M. T. (2009). A single amino acid replacement in ETC2 shapes trichome patterning in natural Arabidopsis populations. Curr. Biol. 19, 1747–1751 10.1016/j.cub.2009.08.057 19818620 PMC2864576

[B16] Hülskamp M. Misra S. Jürgens G. (1994). Genetic dissection of trichome cell development in *Arabidopsis*. Cell 76, 555–566 10.1016/0092-8674(94)90118-X 8313475

[B17] Hülskamp M. Schnittger A. (1998). Spatial regulation of trichome formation in *Arabidopsis thaliana*. Semin. Cell Dev. Biol. 9, 213–220 10.1006/scdb.1997.0209 9599418

[B18] Ishida T. Kurata T. Okada K. Wada T. (2008). A genetic regulatory network in the development of trichomes and root hairs. Annu. Rev. Plant Biol. 59, 364–386 10.1146/annurev.arplant.59.032607.092949 18257710

[B19] Gilroy S1. Jones D. L. (2000). Through form to function: root hair development and nutrient uptake. Trends Plant Sci. 5, 56–60 10.1016/S1360-1385(99)01551-4 10664614

[B20] Kang Y. H. Kirik V. Hulskamp M. Nam K. H. Hagely K. Lee M. M. (2009). The MYB23 gene provides a positive feedback loop for cell fate specification in the Arabidopsis root epidermis. Plant Cell 21, 1080–1094 10.1105/tpc.108.063180 19395683 PMC2685616

[B21] Kang Y. H. Song S. K. Schiefelbein J. Lee M. M. (2013). Nuclear trapping controls the position-dependent localization of CAPRICE in the root epidermis of Arabidopsis. Plant Physiol. 163, 193–204 10.1104/pp.113.221028 23832626 PMC3762640

[B22] Katiyar A. Smita S. Lenka S. K. Rajwanshi R. Chinnusamy V. Bansal K. C. (2012). Genome-wide classification and expression analysis of MYB transcription factor families in rice and Arabidopsis. BMC Genomics 13:544 10.1186/1471-2164-13-544 23050870 PMC3542171

[B23] Kim H. J. Triplett B. A. (2001). Cotton fibre growth in planta and *in vitro*: models for plant cell elongation and cell wall biogenesis. Plant Physiol. 127, 1361–1366 10.1104/pp.010724 11743074 PMC1540163

[B24] Kirik V. Simon M. Hülskamp M. Schiefelbein J. (2004a). The *ENHANCER OF TRY AND CPC1* gene acts redundantly with *TRIPTYCHON* and *CAPRICE* in trichome and root hair cell patterning in *Arabidopsis*. Dev. Biol. 268, 506–513 10.1016/j.ydbio.2003.12.037 15063185

[B25] Kirik V. Simon M. Wester K. Schiefelbein J. Hülskamp M. (2004b). *ENHANCER* of *TRY* and *CPC 2*(*ETC2*) reveals redundancy in the region-specific control of trichome development of *Arabidopsis*. Plant Mol. Biol. 55, 389–398 10.1007/s11103-004-0893-8 15604688

[B26] Koshino-Kimura Y. Wada T. Tachibana T. Tsugeki R. Ishiguro S. Okada K. (2005). Regulation of CAPRICE transcription by MYB proteins for root epidermis differentiation in Arabidopsis. Plant Cell Physiol. 46, 817–826 10.1093/pcp/pci096 15795220

[B27] Kurata T. Ishida T. Kawabata-Awai C. Noguchi M. Hattori S. Sano R. (2005). Cell-to-cell movement of the CAPRICEprotein in Arabidopsis root epidermal cell differentiation. Development 132, 5387–5398 10.1242/dev.02139 16291794

[B28] Kwak S. H. Schiefelbein J. (2007). The role of the SCRAMBLED receptor-like kniase in patterning the Arabidopsis root epidermis. Dev. Biol. 302, 118–131 10.1016/j.ydbio.2006.09.009 17027738

[B29] Kwak S. H. Shen R. Schiefelbein J. (2005). Positional signaling mediated by a receptor-like kinase in Arabidopsis. Science 307, 1111–1113 10.1126/science.1105373 15618487

[B30] Lee M. M. Schiefelbein J. (1999). WEREWOLF, a MYB-related protein in *Arabidopsis*, is a position-dependent regulator of epidermal cell patterning. Cell 99, 473–483 10.1016/S0092-8674(00)81536-6 10589676

[B31] Liu C. Zhao Y. Wang Y. (2006). Artemisinin: current state and perspectives for biotechnological production of an anti malarial drug. Appl. Microbiol. Biotechnol. 72, 11–20 10.1007/s00253-006-0452-0 16773335

[B32] Lommen W. J. Schenk E. Bouwmeester H. J. Verstappen F. W. (2006). Trichome dynamics and artemisinin accumulation during development and senescence of *Artemisia annua* leaves. Planta Med. 72, 336–345 10.1055/s-2005-916202 16557475

[B33] Marks M. D. (1997). Molecular genetic analysis of trichome development in Arabidopsis. Annu. Rev. Plant Physiol. Plant Mol. Biol. 48, 137–163 10.1146/annurev.arplant.48.1.137 15012260

[B34] Masucci J. D. Rerie W. G. Foreman D. R. Zhang M. Galway M. E. Marks M. D. (1996). The homeobox gene *GLABRA2* is required for position-dependent cell differentiation in the root epidermis of *Arabidopsis thaliana*. Development 122, 1253–1260 8620852 10.1242/dev.122.4.1253

[B35] Matsui K. Umemura Y. Ohme-Takagi M. (2008). AtMYBL2, a protein with a single MYB domain, acts as a negative regulator of anthocyanin biosynthesis in Arabidopsis. Plant J. 55, 954–967 10.1111/j.1365-313X.2008.03565.x 18532977

[B36] Mauricio R. Rausher M. D. (1997). Experimental manipulation of putative selective agents provides evidence for the role of natural enemies in the evolution of plant defense. Evolution 51, 1435–1444 10.2307/2411196 28568624

[B37] Morohashi K. Zhao M. Yang M. Read B. Lloyd A. Lamb R. (2007). Participation of the Arabidopsis bHLH factor GL3 in trichome initiation regulatory events. Plant Physiol. 145, 736–746 10.1104/pp.107.104521 17885086 PMC2048771

[B38] Nadeau J. A. Sack F. D. (2002). Control of stomatal distribution on the Arabidopsis leaf surface. Science 296, 1697–1700 10.1126/science.1069596 12040198

[B39] Nemie-Feyissa D. Olafsdottir S. M. Heidari B. Lillo C. (2014). Nitrogen depletion and small R3-MYB transcription factors affecting anthocyanin accumulation in Arabidopsis leaves. Phytochemistry 98, 34–40 10.1016/j.phytochem.2013.12.006 24388610

[B40] Nukumizu Y. Wada T. Tominaga-Wada R. (2013). Tomato (*Solanum lycopersicum*) homologs of TRIPTYCHON (SlTRY) and GLABRA3 (SlGL3) are involved in anthocyanin accumulation. Plant Signal. Behav. 8, e24575 10.4161/psb.24575 23603939 PMC3907391

[B41] Oppenheimer D. G. Herman P. L. Sivakumaran S. Esch J. Marks M. D. (1991). A myb gene required for leaf trichome differentiation in Arabidopsis is expressed in stipules. Cell 67, 483–493 10.1016/0092-8674(91)90523-2 1934056

[B42] Patra B. Pattanaik S. Yuan L. (2013). Ubiquitin protein ligase 3 mediates the proteasomal degradation of GLABROUS 3 and ENHANCER OF GLABROUS 3, regulators of trichome development and flavonoid biosynthesis in Arabidopsis. Plant J. 74, 435–447 10.1111/tpj.12132 23373825

[B43] Payne C. T. Zhang F. Lloyd A. M. (2000). GL3 encodes a bHLH protein that regulates trichome development in Arabidopsis through interaction with GL1 and TTG1. Genetics 156, 1349–1362 11063707 10.1093/genetics/156.3.1349 PMC1461316

[B44] Pesch M. Hülskamp M. (2004). Creating a two-dimensional pattern de novo during Arabidopsis trichome and root hair initiation. Curr. Opin. Genet. Dev. 14, 422–427 10.1016/j.gde.2004.06.007 15261659

[B45] Pesch M. Hülskamp M. (2009). One, two, three...models for trichome patterning in Arabidopsis? Curr. Opin. Plant Biol. 12, 587–592 10.1016/j.pbi.2009.07.015 19783469

[B46] Pesch M. Hülskamp M. (2011). Role of TRIPTYCHON in trichome patterning in Arabidopsis. BMC Plant Biol. 11:130 10.1186/1471-2229-11-130 21951724 PMC3196707

[B47] Pesch M. Schultheiß I. Digiuni S. Uhrig J. F. Hülskamp M. (2013). Mutual control of intracellular localisation of the patterning proteins AtMYC1, GL1 and TRY/CPC in Arabidopsis. Development 140, 3456–3467 10.1242/dev.094698 23900543

[B48] Rerie W. G. Feldmann K. A. Marks M. D. (1994). The *GLABRA2* gene encodes a homeo domain protein required for normal trichome development in *Arabidopsis*. Genes Dev. 8, 1388–1399 10.1101/gad.8.12.1388 7926739

[B49] Ryu K. H. Kang Y. H. Park Y. H. Hwang I. Schiefelbein J. Lee M. M. (2005). The WEREWOLF MYB protein directly regulates CAPRICE transcription during cell fate specification in the Arabidopsis root epidermis. Development 132, 4765–4775 10.1242/dev.02055 16207757

[B50] Ryu K. H. Zheng X. Huang L. Schiefelbein J. (2013). Computational modeling of epidermal cell fate determination systems. Curr. Opin. Plant Biol. 16, 5–10 10.1016/j.pbi.2012.12.003 23287386

[B51] Savage N. S. Walker T. Wieckowski Y. Schiefelbein J. Dolan L. Monk N. A. (2008). A mutual support mechanism through intercellular movement of CAPRICE and GLABRA3 can pattern the Arabidopsis root epidermis. PLoS Biol. 6:e235 10.1371/journal.pbio.0060235 18816165 PMC2553841

[B52] Schellmann S. Hülskamp M. Uhrig J. (2007). Epidermal pattern formation in the root and shoot of Arabidopsis. Biochem. Soc. Trans. 35, 146–148 10.1042/BST0350146 17233622

[B53] Schellmann S. Schnittger A. Kirik V. Wada T. Okada K. Beermann A. (2002). TRIPTYCHON and CAPRICE mediate lateral inhibition during trichome and root hair patterning in *Arabidopsis*. EMBO J. 21, 5036–5046 10.1093/emboj/cdf524 12356720 PMC129046

[B54] Schiefelbein J. (2003). Cell-fate specification in the epidermis: a common patterning mechanism in the root and shoot. Curr. Opin. Plant Biol. 6, 74–78 10.1016/S136952660200002X 12495754

[B55] Schiefelbein J. Huang L. Zheng X. (2014). Regulation of epidermal cell fate in Arabidopsis roots: the importance of multiple feedback loops. Front. Plant Sci. 5:47 10.3389/fpls.2014.00047 24596575 PMC3925829

[B56] Schiefelbein J. Kwak S. H. Wieckowski Y. Barron C. Bruex A. (2009). The gene regulatory network for root epidermal cell-type pattern formation in Arabidopsis. J. Exp. Bot. 60, 1515–1521 10.1093/jxb/ern339 19174459 PMC10941335

[B57] Schiefelbein J. W. (2000). Constructing a plant cell. The genetic control of root hair development. Plant Physiol. 124, 1525–1531 10.1104/pp.124.4.1525 11115870 PMC1539308

[B58] Schnittger A. Folkers U. Schwab B. Jürgens G. Hülskamp M. (1999). Generation of a spacing pattern: the role of *TRIPTYCHON* in trichome patterning in Arabidopsis. Plant Cell 11, 1105–1116 10368181 10.1105/tpc.11.6.1105 PMC144244

[B59] Serna L. (2005). Epidermal cell patterning and differentiation throughout the apical-basal axis of the seedling. J. Exp. Bot. 56, 1983–1989 10.1093/jxb/eri213 15967776

[B60] Serna L. (2008). CAPRICE positively regulates stomatal formation in the Arabidopsis hypocotyl. Plant Signal. Behav. 3, 1077–1082 10.4161/psb.3.12.6254 19513241 PMC2634462

[B61] Simon M. Lee M. M. Lin Y. Gish L. Schiefelbein J. (2007). Distinct and overlapping roles of single-repeat MYB genes in root epidermal patterning. Dev. Biol. 311, 566–578 10.1016/j.ydbio.2007.09.001 17931617

[B62] Song S. K. Ryu K. H. Kang Y. H. Song J. H. Cho Y. H. Yoo S. D. (2011). Cell fate in the Arabidopsis root epidermis is determined by competition between WEREWOLF and CAPRICE. Plant Physiol. 157, 1196–1208 10.1104/pp.111.185785 21914815 PMC3252147

[B63] Stracke R. Werber M. Weisshaar B. (2001). The R2R3-MYB gene family in *Arabidopsis thaliana*. Curr. Opin. Plant Biol. 4, 447–456 10.1016/S1369-5266(00)00199-0 11597504

[B64] Tominaga R. Iwata M. Sano R. Inoue K. Okada K. Wada T. (2008). Arabidopsis CAPRICE-LIKE MYB 3 (CPL3) controls endoreduplication and flowering development in addition to trichome and root hair formation. Development 135, 1335–1345 10.1242/dev.017947 18305006

[B65] Tominaga-Wada R. Ishida T. Wada T. (2011). New insights into the mechanism of development of Arabidopsis root hairs and trichomes. Int. Rev. Cell Mol. Biol. 286, 67–106 10.1016/B978-0-12-385859-7.00002-1 21199780

[B66] Tominaga-Wada R. Nukumizu Y. (2012). Expression analysis of an R3-Type MYB transcription factor CPC-LIKE MYB4 (TRICHOMELESS2) and CPL4-related transcripts in Arabidopsis. Int. J. Mol. Sci. 13, 3478–3491 10.3390/ijms13033478 22489163 PMC3317723

[B67] Tominaga-Wada R. Nukumizu Y. Sato S. Wada T. (2013a). Control of plant trichome and root-hair development by a tomato (*Solanum lycopersicum*) R3 MYB transcription factor. PLoS ONE 8:e54019 10.1371/journal.pone.0054019 23326563 PMC3543402

[B68] Tominaga-Wada R. Nukumizu Y. Wada T. (2013b). Flowering is delayed by mutations in homologous genes CAPRICE and TRYPTICHON in the early flowering Arabidopsis cpl3 mutant. J. Plant Physiol. 170, 1466–1468 10.1016/j.jplph.2013.05.013 23796522

[B69] Wada T. Kurata T. Tominaga R. Koshino-Kimura Y. Tachibana T. Goto K. (2002). Role of a positive regulator of root hair development, *CAPRICE*, in *Arabidopsis* root epidermal cell differentiation. Development 129, 5409–5419 10.1242/dev.00111 12403712

[B70] Wada T. Tachibana T. Shimura Y. Okada K. (1997). Epidermal cell differentiation in *Arabidopsis* determined by a Myb homolog, CPC. Science 277, 1113–1116 10.1126/science.277.5329.1113 9262483

[B71] Wagner G. J. Wang E. Shepherd R. W. (2004). New approaches for studying and exploiting an old protuberance, the plant trichome. Ann. Bot. 93, 3–11 10.1093/aob/mch011 14678941 PMC4242265

[B72] Walker A. R. Davison P. A. Bolognesi-Winfield A. C. James C. M. Srinivasan N. Blundell T. L. (1999). The *TRANSPARENT TESTA GLABRA1* locus, which regulates trichome differentiation and anthocyanin biosynthesis in *Arabidopsis*, encodes a WD40 repeat protein. Plant Cell 11, 1337–1350 10402433 10.1105/tpc.11.7.1337 PMC144274

[B73] Wang S. Barron C. Schiefelbein J. Chen J. G. (2010). Distinct relationships between GLABRA2 and single-repeat R3 MYB transcription factors in the regulation of trichome and root hair patterning in Arabidopsis. New Phytol. 185, 387–400 10.1111/j.1469-8137.2009.03067.x 19878461

[B74] Wang S. Hubbard L. Chang Y. Guo J. Schiefelbein J. Chen J. G. (2008). Comprehensive analysis of single-repeat R3 MYB proteins in epidermal cell patterning and their transcriptional regulation in *Arabidopsis*. BMC Plant Biol. 8:81 10.1186/1471-2229-8-81 18644155 PMC2492867

[B75] Wang S. Kwak S. H. Zeng Q. Ellis B. E. Chen X. Y. Schiefelbein J. (2007). TRICHOMELESS1 regulates trichome patterning by suppressing *GLABRA1* in *Arabidopsis*. Development 134, 3873–3882 10.1242/dev.009597 17933793

[B76] Werker E. (2000). Trichome diversity and development. Adv. Bot. Res. 31, 1–35 10.1016/S0065-2296(00)31005-9

[B77] Wester K. Digiuni S. Geier F. Timmer J. Fleck C. Hülskamp M. (2009). Functional diversity of R3 single-repeat genes in trichome development. Development 136, 1487–1496 10.1242/dev.021733 19336467

[B78] Yu N. Cai W. J. Wang S. Shan C. M. Wang L. J. Chen X. Y. (2010). Temporal control of trichome distribution by MicroRNA156-targeted *SPL* genes in *Arabidopsis thaliana*. Plant Cell 22, 2322–2335 10.1105/tpc.109.072579 20622149 PMC2929091

[B79] Zhang F. Gonzalez A. Zhao M. Payne C. T. Lloyd A. (2003). A network of redundant bHLH proteins functions in all TTG1-dependent pathways of Arabidopsis. Development 130, 4859–4869 10.1242/dev.00681 12917293

[B80] Zhao M. Morohashi K. Hatlestad G. Grotewold E. Lloyd A. (2008). The TTG1-bHLH-MYB complex controls trichome cell fate and patterning through direct targeting of regulatory loci. Development 135, 1991–1999 10.1242/dev.016873 18434419

[B81] Zhu H. F. Fitzsimmons K. Khandelwal A. Kranz R. G. (2009). CPC, a single-repeat R3 MYB, is a negative regulator of anthocyanin biosynthesis in Arabidopsis. Mol. Plant 2, 790–802 10.1093/mp/ssp030 19825656

[B82] Zimmermann I. M. Heim M. A. Weisshaar B. Uhrig J. F. (2004). Comprehensive identification of *Arabidopsis thaliana* MYB transcription factors interacting with R/B-like BHLH proteins. Plant J. 40, 22–34 10.1111/j.1365-313X.2004.02183.x 15361138

