# Supplementary material for: Regulation of cell fate determination by single-repeat R3 MYB transcription factors in Arabidopsis
Source: Front Plant Sci. 2014 Apr 8;5:133. doi: 10.3389/fpls.2014.00133 (PMC3986520; doi:10.3389/fpls.2014.00133)
Supplement: Figure S1 — Amino acid sequence alignment of R3 MYBs with other MYBs. Identical and similar amino acids are shaded in black and gray, respectively. The amino acids of [D/E]Lx2[R/K]x3Lx6Lx3R signature that is required for interaction between MYBs and R/B-like bHLH transcription factors are indicated by red arrowheads on the top of amino acids. The amino acids of WxM motif that is crucial for cell-to-cell movement are indicated by blue arrowhead on the top of amino acids. The pattern of primary structure of MYB repeat, Mx18Wx18W, is indicated by arrows on the bottom of amino acids. [file Presentation1.PDF]

Supplementary Material

Regulation of Cell Fate Determination by Single-repeat R3 MYB Transcription Factors in Arabidopsis

Shucai Wang[1] and Jin-Gui Chen[2, *]

[1] Key Laboratory of Molecular Epigenetics of MOE and Institute of Genetics & Cytology, Northeast Normal University, Changchun, China
[2] Biosciences Division, Oak Ridge National Laboratory, Oak Ridge, TN, USA

* Correspondence:
Biosciences Division
Oak Ridge National Laboratory
1 Bethel Valley Rd
Oak Ridge, TN 37831
USA
Email: chenj@ornl.gov

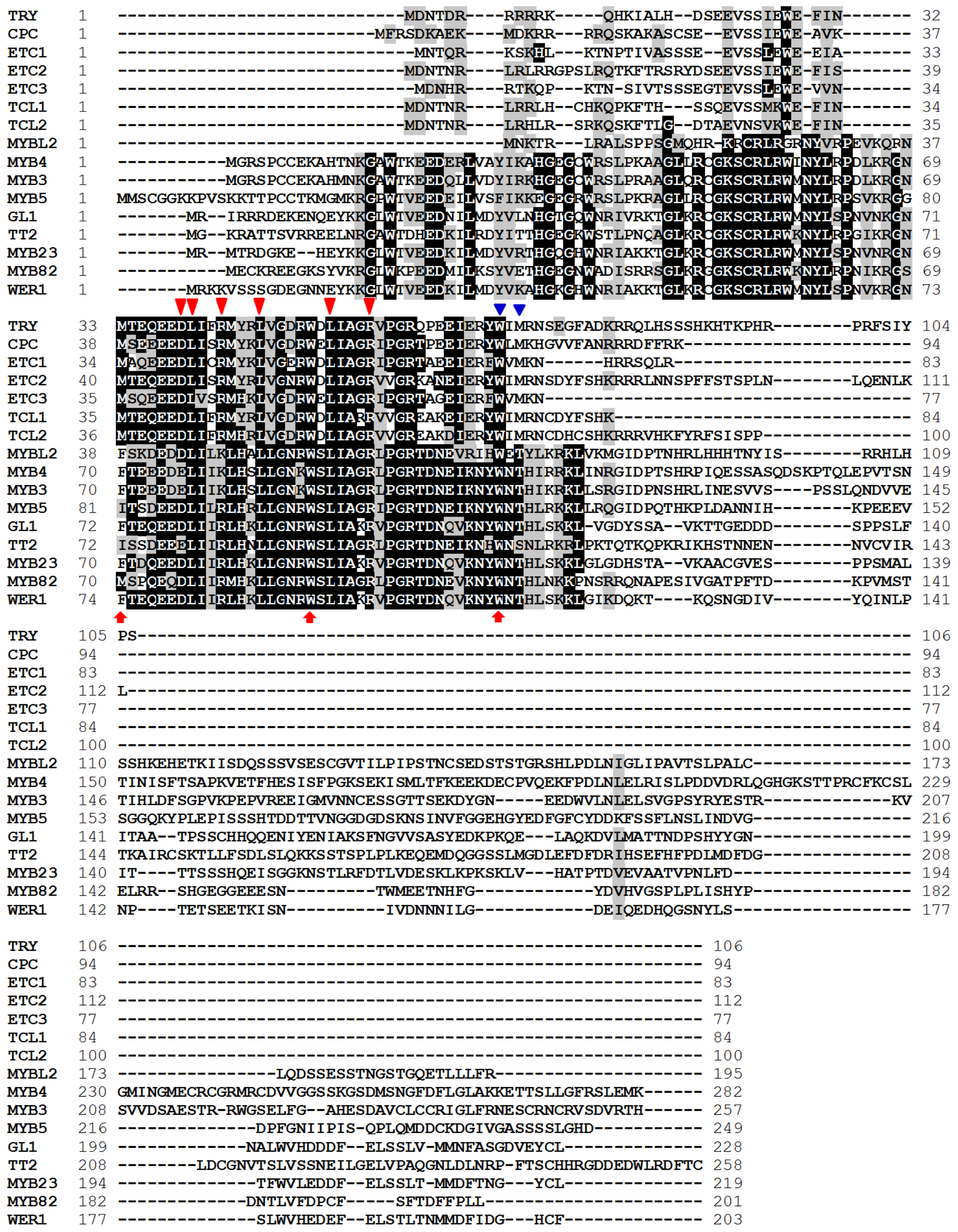

Supplemental Figure S1. Amino acid sequence alignment of R3 MYBs with other MYBs. Identical and similar amino acids are shaded in black and gray respectively. The amino acids of [D/E]Lx_2[R/K]x_3Lx_6Lx_3R signature that is required for interaction between MYBs and R/B-like bHLH transcription factors are indicated by red arrowheads on the top of amino acids. The amino acids of WxM motif that is crucial for cell-to-cell movement are indicated by blue arrowhead on the top of amino acids. The pattern of primary structure of MYB repeat, Mx_{18}Wx_{18}W, is indicated by arrows on the bottom of amino acids.

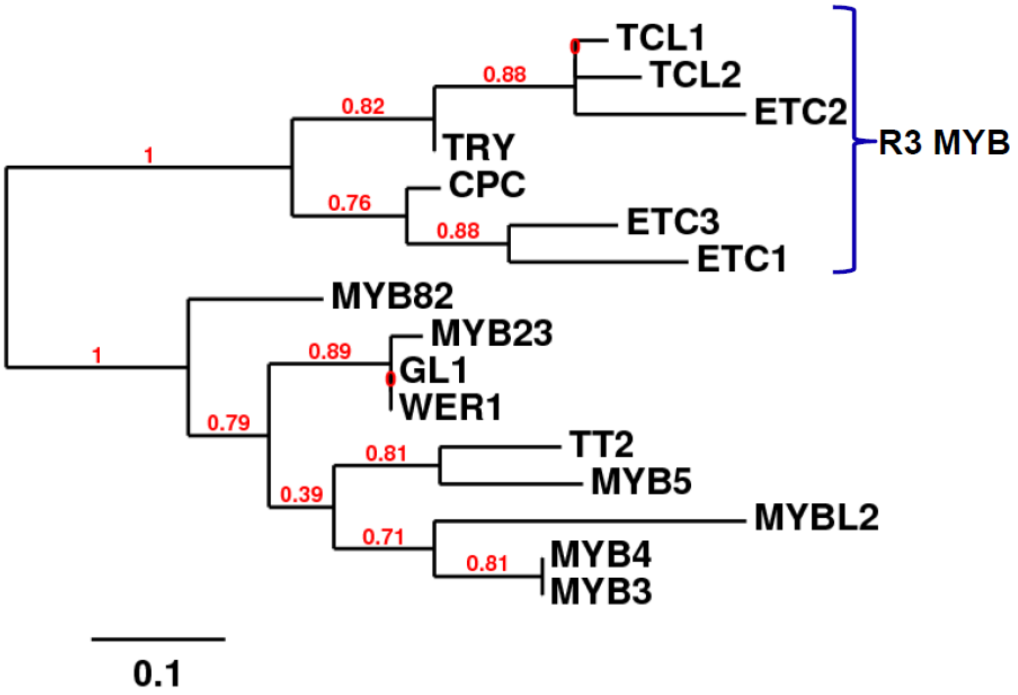

Supplemental Figure S2. Phylogenetic analysis of R3 MYBs with other MYBs. The entire amino acid sequences of single repeat R3 MYB transcription factors were used for phylogenetic analysis using tool on www.phylogeny.fr with default settings.

	TRY	CPC	ETC1	ETC2	ETC3	TCL1	TCL2	MYBL2	MYB4	MYB3	MYB5	GL1	TT2	MYB23	MYB82	WER1
**TRY**		48.6	4504	60.2	40.7	57.4	60.6	21.9	14.9	15.6	17.3	19.3	15.9	19.1	23.3	18.7
**CPC**	67.0		54.3	46.1	52.5	46.3	42.2	16.0	15.9	15.1	17.6	17.8	15.1	17.7	20.9	18.1
**ETC1**	58.5	67.0		40.7	65.9	51.8	39.0	16.3	9.9	11.7	13.9	13.6	13.2	13.7	18.9	15.3
**ETC2**	74.1	62.5	57.1		36.6	59.8	62.5	23.4	14.2	14.8	17.7	18.8	16.7	17.8	20.9	18.1
**ETC3**	56.6	66.0	81.9	51.8		50.0	38.0	13.8	10.6	10.9	13.3	14.5	12.4	13.7	17.4	13.8
**TCL1**	65.1	66.0	69.0	66.1	71.4		68.0	15.7	11.7	11.7	14.5	14.2	12.8	14.5	14.9	15.8
**TCL2**	74.5	68.0	62.0	75.0	59.0	74.0		18.6	13.1	13.2	17.7	15.4	14.7	18.3	16.9	16.3
**MYBL2**	32.8	28.2	24.6	34.4	21.5	26.2	31.3		29.8	31.0	29.7	26.9	27.4	27.6	27.1	26.7
**MYB4**	22.0	22.7	18.1	24.8	17.4	18.1	19.9	44.7		52.1	39.7	31.4	34.6	34.4	31.0	33.2
**MYB3**	24.1	23.7	19.5	26.1	17.5	20.6	21.8	47.9	62.4		38.0	34.0	36.9	35.1	35.4	35.2
**MYB5**	26.5	25.3	20.1	28.5	19.7	21.3	27.3	46.2	56.7	58.8		34.8	37.5	38.2	38.6	40.2
**GL1**	27.2	25.9	20.6	29.4	21.1	21.9	25.0	42.5	50.7	50.2	50.2		33.6	63.8	40.3	55.6
**TT2**	27.5	26.0	19.0	25.6	19.4	22.1	26.7	42.6	54.6	58.5	54.7	49.6		35.5	36.3	35.5
**MYB23**	27.4	24.7	21.5	29.7	21.9	24.7	26.5	44.3	50.0	49.8	53.8	77.2	51.6		43.7	58.9
**MYB82**	31.3	29.9	27.4	34.3	25.9	27.4	30.8	48.8	42.9	49.8	52.6	55.3	48.1	59.4		43.8
**WER1**	31.0	29.1	23.6	30.0	23.6	28.6	24.6	49.8	47.5	50.6	56.2	68.4	50.0	73.1	63.1	

Supplemental Figure S3. Amino acid similarity and identity of R3 MYBs and other MYBs.

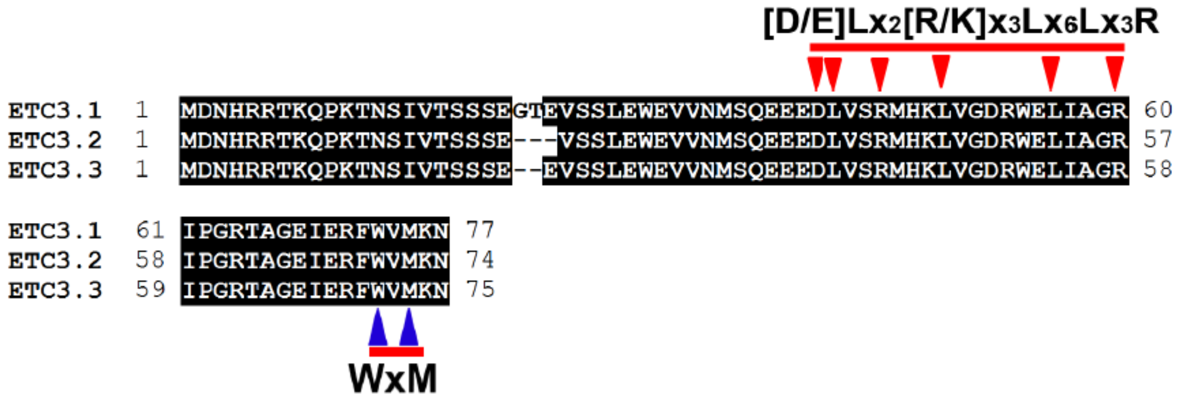

Supplemental Figure S4. Amino acid sequence alignment of ETC3 variants. Identical and similar amino acids are shaded in black and gray, respectively. The amino acids of [D/E]Lx_2[R/K]x_3Lx_6Lx_3R signature that is required for interaction between MYBs and R/B-like bHLH transcription factors are indicated by arrowheads on the top of amino acids. The amino acids of WxM motif that is crucial for cell-to-cell movement are indicated by arrowheads on the bottom of amino acids.
